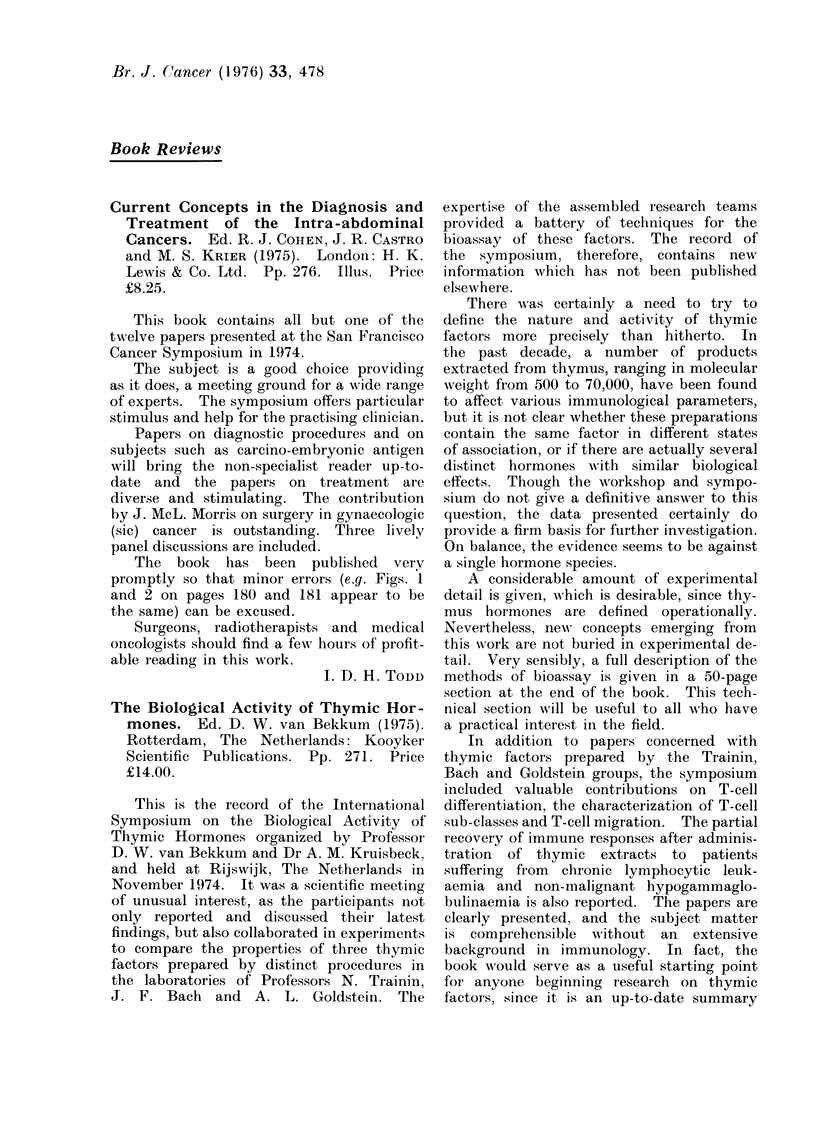# Current Concepts in the Diagnosis and Treatment of the Intra-abdominal Cancers

**Published:** 1976-04

**Authors:** I. D. H. Todd


*Br. J. Cancer* (1976) **33**, 478

### Book Reviews

**Current Concepts in the Diagnosis and Treatment of the Intra-abdominal Cancers.** Ed. R. J. COHEN, J. R. CASTRO and M. S. KRIER (1975). London: H. K. Lewis & Co. Ltd. Pp. 276. Illus. Price £8.25.

This book contains all but one of the twelve papers presented at the San Francisco Cancer Symposium in 1974.

The subject is a good choice providing as it does, a meeting ground for a wide range of experts. The symposium offers particular stimulus and help for the practising clinician.

Papers on diagnostic procedures and on subjects such as carcino-embryonic antigen will bring the non-specialist reader up-to-date and the papers on treatment are diverse and stimulating. The contribution by J. McL. Morris on surgery in gynaecologic (sic) cancer is outstanding. Three lively panel discussions are included.

The book has been published very promptly so that minor errors (*e.g.* Figs. 1 and 2 on pages 180 and 181 appear to be the same) can be excused.

Surgeons, radiotherapists and medical oncologists should find a few hours of profitable reading in this work.

I. D. H. TODD